# (Update of a) systematic review on the impact of elective early term (< 39th gestational week) caesarean sections on maternal and neonatal health - a protocol

**DOI:** 10.1186/s13643-018-0787-5

**Published:** 2018-08-16

**Authors:** Barbara Prediger, Stephanie Polus, Tim Mathes, Stefanie Bühn, Frank Louwen, Edmund A. M. Neugebauer, Dawid Pieper

**Affiliations:** 1 Institute for Research in Operative Medicine, Witten/Herdecke University, Ostmerheimer Str. 200, Haus 38, 51109 Cologne, Germany; 2 Department of Gynecology and Obstetrics, Goethe-University, Theodor-Stern-Kai 7, 60590 Frankfurt, Germany; 3 Brandenburg Medical School - Theodor Fontane, Faculty of Health, Campus Neuruppin, Fehrbelliner Str.38, 16816 Neuruppin, Germany; 4 Interdisciplinary Centre for Health Services Research, Witten/Herdecke University, Alfred-Herrhausen-Straße 50, 58448 Witten, Germany

**Keywords:** Elective caesarean section, Term birth, Neonatal intensive care unit, Neonatal morbidity, Maternal morbidity, Gestational age

## Abstract

**Background:**

The rate of caesarean sections increased in the last decades to about 30% of births in western populations. Many caesarean sections are electively planned without an urgent medical reason for mother or child. Especially in women with a foregoing caesarean section, the procedure is planned early. An early caesarean section though may harm the newborn. Our aim is to evaluate the gestational time point after the 37th gestational week (after prematurity = term) of performing an elective caesarean section with the lowest morbidity for mother and child.

**Methods:**

This is an update of a systematic review previously carried out on behalf of the German Federal Ministry of Health. We will perform a systematic literature search in MEDLINE, EMBASE, CENTRAL and CINAHL. Our primary outcome is the rate of admissions to the neonatal intensive care unit in early versus late term neonates. We will include (quasi) randomized controlled trials and cohort studies. The studies should include pregnant women who have an elective caesarean section at term. We will screen titles and abstracts and the identified full texts of studies for eligibility. Risk of bias will be assessed with the Cochrane Risk of Bias Tool for Randomized Trials or with the Risk of Bias Tool for Non-Randomized Studies of Interventions (ROBINS-I). These tasks will be performed independently by two reviewers. Data will be extracted in beforehand piloted extraction tables. A dose-response meta-analysis will be performed.

**Discussion:**

Our aim is to reach a higher validity in the assessment of the time point of elective caesarean sections by performing a meta-analysis to support recommendations for clinical practice. We assume to identify less randomized controlled trials but a large number of cohort studies analyzing the given question. We will discuss similarities and differences in included studies as well as methodological strengths and weaknesses.

**Systematic review registration:**

PROSPERO CRD42017078231

## Background

In the last decades, the rates of caesarean section in western countries have increased to about 30% of all births [1]. The World Health Organization (WHO) states that there is no medical reason for a higher rate of caesarean sections than 10–15%, though [2, 3]. One can assume a high number of caesarean sections planned electively without an urgent medical need in women or children. It is in recent discussion when the optimal time point of performing an elective caesarean section could be. According to an analysis of 63 English NHS trusts in which women were undergoing elective caesarean sections, 97% of elective caesarean sections are performed beyond 37th + 0 gestational week [4]. There are two guidelines “Caesarean Section” by NICE and “Birth after previous caesarean birth” by the Royal College for Obstetricians & Gynaecologists which support the thesis that the later the section is performed, the risk for respiratory morbidity of the newborn decreases [5, 6]. The NICE guideline was first published in 2004. The analysis of the NHS trusts showed an increase of caesarean sections ≥ 39th gestational week from 39% in 2000 to 63% in 2009 [4].

For a development in childbirth towards more electively planned caesarean sections, it is essential to provide a time point for the caesarean section with the lowest risk for mother and child.

The most common reason for performing an elective caesarean section is a previous caesarean section. Even though vaginal birth after caesarean section (VBAC) is recommended for the majority of women, a recent study showed that in the UK, only 50% of women with previous caesarean section would undergo VBAC in English NHS trusts [7]. In the USA, the rate of VBAC was only about 10% in 2007 [8].

Women with a scarred uterus may have diverse risks in following pregnancies. Especially with the growing unborn in the last weeks of pregnancy, the risk of scar rupture may increase [9]. A higher risk of bleeding needing transfusion and injuries at the bladder is assumed. Even higher mortality rate might be connected to a late term elective caesarean. These risks do not touch women without prior caesarean section/intact uterus. But labor can occur earlier than the planned time point of caesarean section, and this may result in an emergency caesarean section [10]. These assumptions lead to a practice of early term elective caesareans.

On the other hand, the newborn even though at term (37th pregnancy week) is under risk for various disorders. Especially respirational severities are linked to an early term caesarean section. Lungs are mature in 37th gestational week, but neonates born by caesarean have a general higher risk of respiratory disorders [11]. The longer an unborn is kept in the uterus, it gains more weight and becomes more resistant.

On behalf of the German Federal Ministry of Health, a systematic review was performed in 2016 to analyze these concerns [12]. We performed a systematic search in May 2016 in MEDLINE, EMBASE, CENTRAL and CINAHL. Search results were screened and assessed for eligibility by two reviewers independently. Data were extracted in Review Manager 5.3, and risk of bias was assessed in randomized controlled trials (RCT) with the Cochrane risk of bias tool and in cohort studies with the Newcastle-Ottawa Scale (NOS) by two reviewers independently [13, 14]. Meta-analyses were performed for each outcome when study data were not too heterogeneous. Gestational weeks compared were 37th + 0 until 38th + 7 versus ≥ 39th + 0 weeks. We included 30 studies covering 982,425 patients. Except one RCT, we only included cohort studies. The primary outcome NICU admission showed a significant higher rate in the 37th–38th weeks group (relative risk 1.78 [95% CI 1.55, 2.06]). The review is only available in German [12]. Herewith, we will update this review and also will broaden the perspective to an international context. We aim to expand the reach of the findings with this update in English. Moreover, we will perform another meta-analysis which, as we assume, shows a linear time-response relationship.

We aim to analyze with a systematic review of the literature whether:

- There is an increased risk of mortality for mothers with a full term (39th pregnancy week) elective caesarean section compared to early term caesarean section
- There is a decreasing risk for the newborn for mortality and Neonatal Intensive Care Unit (NICU) admission in full term caesarean section compared to early term caesarean section

## Methods/design

### Eligibility criteria

We will include women with a planned caesarean section at term (≥ 37th gestational week), regardless if it is first caesarean or repeated caesarean section. The population will be limited to WHO Stratum A. This covers states with very low child and very low adult mortality including Western Europe, North America and various Western Pacific states [15]. We chose this stratum not only because of the very low general mortality but also because of a comparable caesarean section rate. We did not define any other exclusion criteria regarding to the population. We will include studies with single and multiple pregnancies. If a study includes exclusively multiple pregnancies, we will conduct a subgroup analysis. Our interest is a planned caesarean section at early term (37th–38th week) compared to full term (≥ 39th week). The primary outcomes are neonatal death, neonatal intensive care unit admission and maternal death. For secondary outcomes, see Table 1. Outcomes with unspecific definition like respiratory morbidity or composite adverse events will be reported as it is defined in the relevant study. We will not make any restrictions regarding the language and publication date. If necessary, we will prompt translations of studies in languages unknown to the authors. We will consider RCTs, quasi RCTs and cohort studies. Both recommendations in the NICE and RCOG guidelines are based on cohort studies, and it is to assume that RCTs are exceptional.

**Table 1:** PICOS

Population	Women with electively planned caesarean section beyond prematurity (≥ 37th gestational week), regardless of prior pregnancy or prior birth mode. WHO Stratum A
Intervention	Elective caesarean section at 37th + 0 until 38th + 6
Comparator	Elective caesarean section ≥ 39th + 0
Outcome	Primary outcome:
	Neonatal death, neonatal intensive care unit admission and maternal death
	Secondary outcome:
	Neonatal: intensive care unit length of stay, respiratory morbidity, RDS, TTN, pneumothorax, hypoglycemia*, Apgar score, hyperbilirubinemia needing phototherapy, birth weight and near miss
	Maternal: hysterectomy, bleeding needing transfusion, near miss and adverse events
Study type	RCT, quasi RCT and cohort study

*RCT* randomized controlled trial, *RDS* respiratory distress syndrome, *TTN* Transient tachypnea of the neonate

*Depending on the age at assessment: 0–3 h < 2.0 mmol/l; 3–24 h < 2.2 mmol/l; > 24 h < 2.5 mmol/l [30]

### Information sources

We will systematically search MEDLINE, EMBASE, CENTRAL and CINAHL. Study registries will be searched for new studies (ClinicalTrials, Deutsches Register Klinischer Studien and EU clinical trials register) [BP; SB]. To identify grey literature, we will search in Google Scholar additionally [BP].

We will also check the references in included studies, guidelines and systematic reviews, and if necessary, will contact authors for additional data [BP].

### Search strategy

Search strategy as a draft for MEDLINE:

**Table:** 

(neonatal[tiab] OR neo-natal[tiab] OR maternal[tiab] OR Perinatal[tiab] OR peri-natal[tiab] OR “Perinatal Care”[Mesh] OR “intensive care”[tiab] OR “Intensive Care Units”[Mesh] OR oxygen[tiab] OR bleeding[tiab] OR Apgar[tiab] OR hypoglycemia[tiab] OR “Hypoglycemia”[Mesh] OR .hyperbilirubinemia[tiab] OR “Hyperbilirubinemia, Neonatal”[Mesh] OR “birth weight”[tiab] OR “Birth Weight”[Mesh] OR antibiotic*[tiab] OR respirator*[tiab] OR CPAP[tiab] OR “Continuous Positive Airway Pressure”[Mesh]) AND (Cesarean[tiab] OR Caesarean[tiab] OR Cesarian[tiab] OR Caesarian[tiab] OR “Cesarean Section”[Mesh] OR CSection[tiab] OR “C Section”[tiab] OR “C Sections”[tiab]) AND (Timing[tiab] OR late[tiab] OR prior[tiab] OR delayed[tiab] OR time[tiab] OR week*[tiab]) AND Elective[tiab]	

Search strategy will be developed by DP using MeSH terms and text words, and a librarian will check the strategy by applying the PRESS checklist [16].

### Study selection

Records identified through the searches will be added to an Endnote X7 database, and duplicates will be removed. Two reviewers will independently assess the relevance of the identified titles and abstracts [BP, SP]. Studies which are included for full text review will again be independently assessed by the same two reviewers. Differences will be discussed until a consensus is found or a third reviewer will be included. The process will be completed in the same Endnote database. According to PRISMA, a flowchart of the study selection process will be developed.

### Data collection

Data will be collected in a beforehand piloted abstraction table by one reviewer [BP]; the other reviewer will monitor the entry for completeness and accuracy [SP]. We will extract data directly in a excel sheet. Items will be presented according to Table 2.

**Table 2:** Data extraction

Methods	Study type: RCT, cohort study
	Setting: academic hospital, general clinic…
	Recruitment period:
Participants	Exclusion criteria:
	Characteristics: age, ethnicity, BMI, parity, previous caesarean, smoking status, marital status, maternal disease and indication for caesarean
	Included patients:
	Drop outs:
Time points	Time point of caesarean section:
Outcomes	Primary outcome: neonatal death, neonatal intensive care unit admission and maternal death
	Secondary outcome:
	Neonatal: intensive care unit length of stay, hospitalization, respiratory morbidity, RDS, TTN, pneumothorax, hypoglycemia, Apgar Score, hyperbilirubinemia needing phototherapy, birth weight and near miss
	Maternal: hysterectomy, bleeding needing transfusion near miss and adverse events

### Risk of bias assessment

Risk of bias will be assessed by two reviewers independently [BP, SP]. For (q) RCTs we will use the Cochrane Risk of Bias Tool [13]. In the systematic review performed for the German Federal Ministry of Health, we used the Newcastle-Ottawa Scale (NOS) to assess non-randomized studies, since ROBINS-I Tool was not published at that stage when a protocol was developed for the German Federal Ministry of Health. However, we found that NOS contained a few items to unspecific for the purpose of our review [14]. For cohort studies, we will use the ROBINS-I Tool and assess all new studies as well as reassess all studies discovered in the prior report [17].

### Data synthesis

If studies are sufficiently clinical homogenous, a meta-analysis will be performed. We will contact the authors to receive unadjusted data if only adjusted data are provided in the publication. We will perform a multivariate dose-response meta-analysis for pooling outcomes. Instead of “dose”, we use “time” as variable starting with 37th + 0 gestational week up to 42nd + 0 in weekly steps. According to the results of the systematic review performed for the German Ministry of Health and other published literature, we assume a linear time-response relationship, for neonatal (adverse) outcomes, a regressive and for maternal (adverse) outcomes, a progressive trend is assigned [11, 12, 18]. The analysis will assume a linear time-response relationship. We will examine visually for each outcome if the assumed linear time-response relationship is effectively present. If the relationship deviates from a linear form, we will use fractional polynomials to model a non-linear association [19]. In the first stage, we will estimate a time-response curve (i.e. gestational week-outcome) for each study across gestational week values observed in the whole dataset. In the second stage, these curves will be pooled into an overall gestational week-outcome curve. The time-response analysis will follow the methods by Greenland and Longnecker [20]. We will calculate study-specific slopes (linear trends), 95% confidence intervals from the natural logs of the reported effect measures and confidence intervals across gestational weeks, taking the correlations between RRs into account. In case of the reference category being not the lowest category, we will first try to recalculate data in such a way that the lowest category will be the reference category. In cases where this will not be possible, we will exclude the categories below the reference category for the linear time-response analysis. For studies reporting ranges of weeks, the midpoint of the lower and upper cut-off will be assigned for each category. When upper and lower categories will be open-ended, the lower and upper cut-off value will be 37th + 0 and 42nd + 7 weeks. Again, the midpoint of the lower and upper cut-off will be assigned for each category. When authors report the median or mean per category, this will be used to assign the corresponding RR for each study.

In a sensitivity analysis, we will conduct a univariate random effects meta-analysis (37th + 0–8th + 6 vs ≥ 39th + 0 gestational week). If more than one effect estimate will be reported, we will choose the model with the greatest degree of control for potential confounding. We will calculate pooled risk ratios, mean differences or if necessary, standardized mean differences. We will use the Paule and Mandel heterogeneity variance estimator and modified Hartung-Knapp confidence intervals for the pooled estimates [21, 22]. Beta-binomial models (random effects model) will be computed for rare events, such as mortality [23].

If possible, subgroup analyses will be performed for the following variables:

- Repeat elective caesarean versus first caesarean section.
- Study region (North America, Europe and Asia).
- Maternal age.
- Maternal BMI.
- Multiple pregnancies.

Additionally, we will conduct sensitivity analyses according to study design and dependent on the results of risk of bias assessment.

We will consider clinical and methodological heterogeneity. Therefore, clinical experts will assess clinical heterogeneity and methodologists will assess methodological heterogeneity. Statistical heterogeneity will be assessed by the *Q* test and *I*^2^ statistic [24].

All analyses will be performed with *R* 3.3.2 using the metaphor and dosresmeta packages [25, 26].

If data are too heterogeneous, we will perform a structured narrative analysis of the outcome. We will use GRADE to synthesize the results [27]. Two reviewers (BP and SP) will independently perform the GRADE assessment for each outcome with the GRADEpro GDT software. Domains assessed with the GRADE approach are risk of bias, inconsistency, indirectness, imprecision, publication bias, large effects, confounding and dose-response gradients.

### Meta-biases

#### Publication bias

We will assess publication bias by visual inspection of the funnel plots for asymmetry. Furthermore, we will apply Egger’s test and Begg’s test [28, 29]. A *p* value < 0.1 will be considered statistically significant. Study registers will be searched for (yet) unpublished studies.

#### Selective reporting within studies

If available, study protocols will be checked and compared with reporting in studies. We will ask authors for availability of study protocols and search ClinicalTrials.gov.

## Discussion

This review should cover a broad range of studies comparing an early term with a late term elective caesarean section. We will include RCTs and cohort studies, though we assume that the number of RCTs will be small. The results will be discussed and critically appraised. We are looking for evidence which can support the decision for the time point of elective caesarean section. See Table 3 for the differences between the old (2016) and the update systematic review. This review should improve the outcomes for mother and child in elective caesarean section by being a help in medical decision making.

**Table 3:** Differences between 2016 and update systematic review

	Systematic review on behalf of the German Federal Ministry of Health 2016	Systematic review planned 2017
Language	German	English
Publication	Website of the German Federal Ministry of Health	International peer reviewed journal
Grey literature search	None	Google Scholar
Risk of bias assessment in non-randomized studies	Newcastle-Ottawa Scale	ROBINS-I tool for non-randomized studies
Comparison	Comparison of the 37th–38th pregnancy week to ≥ 39th pregnancy week	Time-response analysis including 37th until 42nd pregnancy week including steps of 1 week
Meta-analysis	Mantel-Haenszel test considering random effects	Time-response analysis, see section data synthesis
Software	Review Manager 5.3	*R* 3.3.2 + metaphor and dosresmeta package
Results	NICU admission (primary outcome): higher rate in the 37th–38th weeks group (relative risk 1.78 [95% CI 1.55, 2.06])	–

## Abbreviations

NICU: Neonatal intensive care unit
RCT: Randomized controlled trial
VBAC: Vaginal Birth after cesarean section
WHO: World Health Organization
